# Insights into the Genetics Underlying the Resistance to Root-Knot Nematode Reproduction in the Common Bean Ouro Negro

**DOI:** 10.3390/plants14071073

**Published:** 2025-04-01

**Authors:** Ana M. Pesqueira, Ana M. González, Teresa Barragán-Lozano, María S. Arnedo, Rafael Lozano, Marta Santalla

**Affiliations:** 1 Grupo de Genética del Desarrollo de Plantas, Misión Biológica de Galicia-CSIC, P.O. Box 28, 36080 Pontevedra, Spain; ampesqueira@mbg.csic.es (A.M.P.); amgonzalez@mbg.csic.es (A.M.G.); 2 Centro de Investigación en Biotecnología Agroalimentaria (CIAIMBITAL), Universidad de Almería, 04120 Almería, Spain; teresa@ual.es (T.B.-L.); rlozano@ual.es (R.L.); 3 Semillas Ramiro Arnedo, Las Norias de Daza, 04716 Almería, Spain; marnedo@ramiroarnedo.com

**Keywords:** *Phaseolus vulgaris*, genetics, resistance, *Meloidogyne*, root-knot nematode

## Abstract

Root-knot nematodes (RKNs, *Meloidogyne* spp.) have become the major yield-limiting biological factor in common bean production in many warmer-climate regions such as the south of Europe. Broadening the genetic base of resistance in elite common bean cultivars is the most effective and environmentally friendly method for managing this disease. Toward this goal, F_1_, F_2_, and F_3_ populations from crosses between susceptible snap beans (Helda and Perona) and the resistant Ouro Negro cultivar were phenotyped for *M. incognita* and *M. javanica*-induced root-galling (GI) and egg mass production (EM) in controlled growth chamber infection assays. F_1_ progenies showed a susceptible response to both RKN isolates, with high GI and EM values, indicating a recessive inheritance of nematode resistance. The estimates for broad-sense heritability for GI and EM in the F_2_ Helda × Ouro Negro population infected with *M. incognita* were 0.62 and 0.54, respectively. RKN resistance in Ouro Negro is largely controlled by partial to overdominant genetic effects and that susceptibility factor leads recessive resistance. The minimum number of genes involved in nematode resistance was estimated to be about two or three. In agreement, genetic analysis of F_2_ segregating populations supported duplicate recessive epistasis as the inheritance pattern involved in the resistance provided by the Ouro Negro cultivar. Ouro Negro is an important resource for broadening RKN resistance in elite common bean cultivars.

## 1. Introduction

Snap bean (*Phaseolus vulgaris* L.) is a warm-season vegetable legume crop harvested for its immature seed pods—they are most tender and succulent before seed maturation causes the pod walls to expand. Snap bean is a catchall term that includes green beans, string beans, yellow wax beans, Italian Romano beans, and French beans. It is the second most important vegetable legume crop: a total mass of 23 million tons was grown over 1 million ha worldwide in 2022 [1]. High yields and a year-round income favor greenhouse vegetable legume production over that in open fields, as more than 80% of the total production is linked to foreign trade. These products produced in greenhouses are mainly grown for the fresh market, and greenhouse vegetable legume production industry is undergoing a rapid expansion in many regions of the world. This crop has many advantages but faces challenges in the three different vegetable markets (fresh, canned, and frozen), including quality requirements, as well as strict control for pesticide residues.

As a result of intensive agriculture and cultivation, the occurrence of root-knot nematodes has increased. They can be found in all horticultural areas [2], causing yield losses in all essential fruit and vegetable crops, such as cucumber (85%), tomato (59%), zucchini (40%), watermelon (36%), lettuce (29%), and common bean (20–90%) [3,4,5]. *Meloidogyne* spp. have been detected worldwide, predominantly in warmer climates. They adapt very well to the environmental conditions used to produce the protected crops, and their life cycle and reproductive strategies make their total eradication from agricultural soils almost impossible. The southern Mediterranean Basin, with mild winters and warm summers, has the highest concentration of horticultural production in Europe, characterized by valuable crops such as the common bean, where *M. arenaria*, *M. incognita*, and *M. javanica* are the most common root-knot nematode (RKN) species causing production losses [6,7,8]. Strategies to control RKNs are complex due to their broad host range, their concealment in soil, the lack of resistant varieties, and the ban on many chemical nematicides because of their high environmental toxicity. Hence, breeders should focus on developing resistant cultivars, which is essential from economical, practical, and environmental perspectives.

A search for high levels of RKN resistance in some vegetable crops has been undertaken in recent years by using complex and laborious phenotyping procedures, and currently, resistant cultivars are commercially available for different horticultural crops such as tomato, pepper, watermelon, and eggplant. However, the majority of commercially cultivated bean cultivars are highly susceptible to the RKN disease caused by *Meloidogyne* spp. [9,10]. Only a limited number of bean cultivars with an acceptable level of RKN resistance have been developed [4,11,12,13], and the Ouro Negro cultivar has been classified as broadly resistant to a great majority of pathogens, including RKN isolates [14,15,16,17]. An additional feature is the presence of genetic variability in the response to RKN due to the geographical origin of the nematode population [18,19,20], particularly to the predominant type of RKN, relative virulence elements, and contributing environmental factors (e.g., temperature). Previous works uncovered the complexity of bean–RKN interactions and highlight the importance of conducting studies aimed at dissecting the genetic architecture of host responses and identifying the genes underlying this resistance for the development of resistant bean cultivars.

Effective RKN resistance genes are broadly available in a wide range of host plant taxa [21]. The identification of effective host plant resistance genes that can be introgressed into crop cultivars constitutes an important component of nematode breeding programs. Nematode resistance genes block or suppress one or more of the critical steps in nematode infection. Different single dominant RKN-resistant genes have been identified in tomato [22,23], pepper [24,25], and peanut [26,27], and some of them have been successfully introgressed in some crops and are extensively used by breeders and farmers [28,29,30]. In the common bean, the genetic control varies depending on the resistance sources considered. There are reports that have identified the recessive *mir-1* gene and two dominant genes, *Mig-1* and *Mjg-1*, in lima bean as controlling the reproduction of *M. incognita* and the galling of *M. incognita* and *M. javanica* [31]. Previous studies on the common bean [13,32] have reported that *M. hapla*, *M. incognita*, and *M. javanica* resistance is controlled by two dominant genes (*Me1*, *Me2*) and one recessive (*me3*) gene under the control of a single locus, with dominance or incomplete dominance for *M. incognita* resistance [33,34,35]. Therefore, this variability regarding the control of RKN resistance for root-galling and nematode reproduction in common bean suggests that additional efforts are needed to clarify the genetic bases underlying resistance.

The common bean cultivar Ouro Negro bears a broad spectrum of resistance genes against anthracnose (*Co-10*), angular leaf spot (*Phg-ON*), and rust (*Ur-14*) [36,37,38], as well as against *M. incognita* and *M. javanica* [14,16,17], but the genetic determinants of this resistance are still not known. The present study aims to decipher the inheritance pattern of resistance to *M. incognita* and *M. javanica* in Ouro Negro and to utilize its resistance as a source of variability for broadening the genetic base of snap bean breeding cultivars. To achieve this, we examined the segregation of root-galling and nematode reproduction in hybrid, F_2_, and F_3_ populations derived from two different crosses. The type of gene action, heterosis values, number of genes, and heritability were estimated, with the results indicating that at least two independent genes contribute to nematode resistance. This study forms the foundation for identifying these resistance genes and provides valuable prebreeding lines that will be of interest to the legume breeding community.

## 2. Results

### 2.1. Response of Common Bean Genotypes to M. incognita and M. javanica Infection

**Figure 1:**
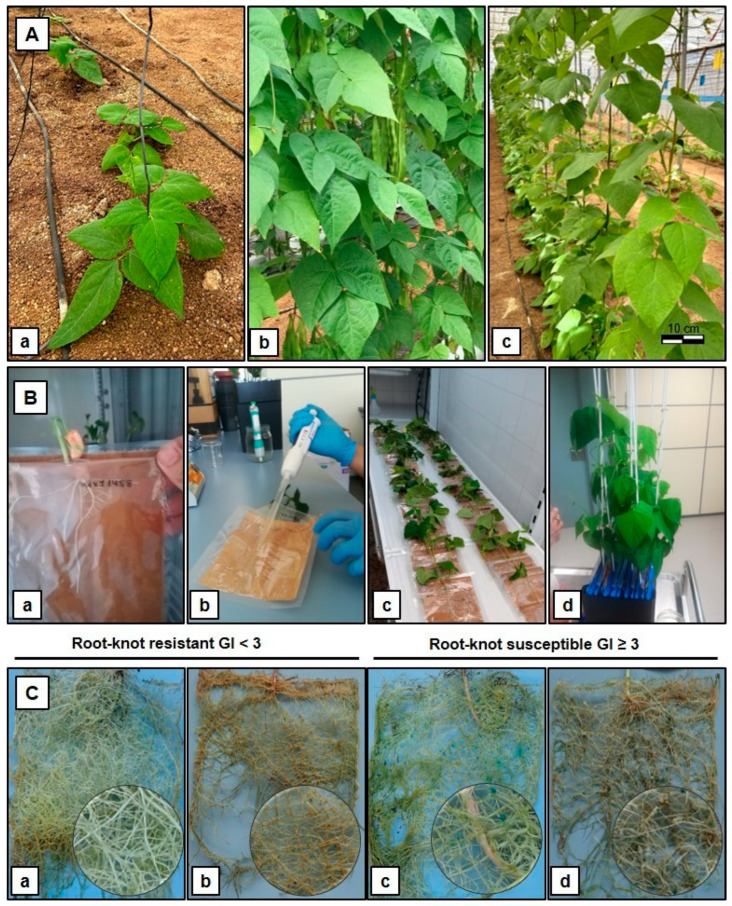
Developmental stages of common bean plants infected with *M. incognita* and *M. javanica* nematode species. (**A**) Non-treated parents growing in greenhouse soil: (**a**) resistant Ouro negro cultivar; (**b**) susceptible Helda; and (**c**) Perona snap breeding lines. (**B**) Root-knot nematode infection process: (**a**) a two-week-old common bean plant grown in a pouch and ready for root-knot nematode inoculation; (**b**) root-knot nematode inoculation with a 5 mL pipette tip; (**c**) the pouch in a horizontal position for 24 h after inoculation; and (**d**) treated plants grown in a growth chamber 21 days post-inoculation. (**C**) A common bean root system with egg masses stained with erioglaucine 45 days post-inoculation: (**a**) a common bean root system evaluated at a root-knot gall index (GI) = 0; (**b**) a root system evaluated at GI = 2; (**c**) a root system evaluated at GI = 4; and (**d**) a root system evaluated at GI = 6. A GI of <3 indicates resistance, and GI ≥ 3 indicates susceptible classes. Bar = 10 cm.

Resistance to the nematode species *M. incognita* and *M. javanica* was analyzed in a set of common bean genotypes, which included two cultivars previously reported to be resistant (Ouro Negro and Aporé) and five susceptible varieties (PhyIM, Phy90, Phy5, Helda, and Perona) (Figure 1A). In addition, F_1_ progenies obtained from Helda × Ouro Negro and Perona × Ouro Negro crosses were analyzed. The resistance response was evaluated based on two criteria (Figure 1B), the galling index (GI), and egg mass number (EM); values are shown in Table 1. As expected, the susceptible cultivars PhyIM, Phy90, and Phy5 generally showed the highest GI and EM values in response to both isolates. In the Ouro Negro and Aporé cultivars, infection with either *M. incognita* or *M. javanica* led to much lower GI and EM values than those obtained for the other genotypes (Figure 1Ca,b). Egg masses and the extent of galling were also observed in the Helda and Perona varieties (Figure 1Cc). Both HO and PO F_1_ hybrids exhibited susceptible values for GI and EM (Figure 1Cd), suggesting recessive inheritance of nematode resistance (Table 1).

**Figure 2:**
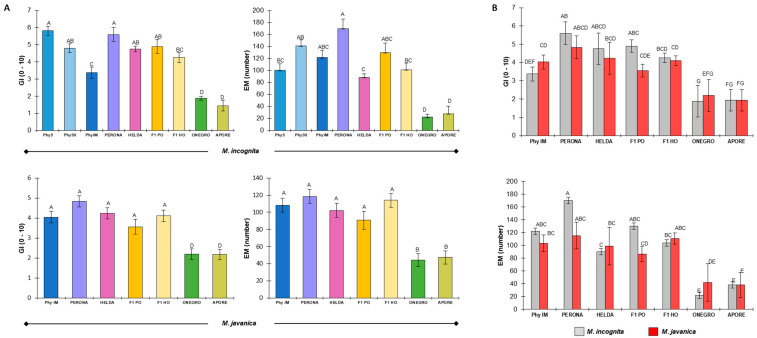
Nematode resistance response of the common bean genotypes and F_1_ progeny from the single crosses of Helda and Perona × Ouro Negro (F_1_ HO and F_1_ PO, respectively) after *M. incognita* and *M. javanica* infection. (**A**) GI and EM values for each common bean genotype with the same nematode isolate. (**B**) GI and EM values for each nematode isolate with the same common bean genotype. Different letters indicate significant differences (*p* < 005; ANOVA and Tukey’s Honestly Significant Difference (HSD) test). The HSD critical values: 4.458 (*M. incognita*) and 4.257 (*M. javanica*) (**A**); 4.325 (**B**). Vertical bars represent standard errors. Galling index (GI), number of egg masses per plant (EM).

The two-way ANOVA showed significant effects for the common bean genotype (*p* < 10^−3^), the *M. incognita* and *M. javanica* isolates (*p* < 10^−3^), and the isolate × genotype interaction (*p* < 10^−2^). The average GI and EM values of the common bean genotypes and F_1_ hybrids were individually compared for each nematode isolate (Figure 2A). Helda, Perona, and F_1_ progenies showed non-significant differences for GI and EM values compared to susceptible controls regardless of the nematode isolate, whereas Ouro Negro and Aporé displayed significantly lower GI and EM values for both isolates. With the use of the same dataset, the mean EM and GI values of the two *Meloydogine* species were compared for each common bean genotype (Figure 2B), and no significant differences were found between the isolates. These results agreed with the analysis of the correlation between *M. incognita* and *M. javanica* data, where GI and EM were highly correlated between isolates (r = 0.80, *p* = 0.0001). Therefore, the *M. javanica* isolate was not included in subsequent statistical analyses of the segregant F_2_ progeny.

### 2.2. Heterosis Performance of F1 Hybrids

**Figure 3:**
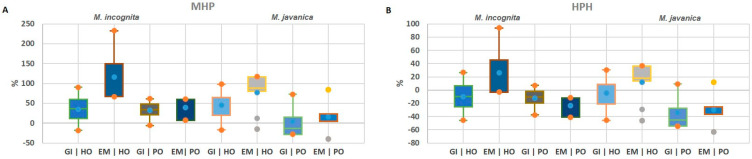
Heterosis performance of F_1_ hybrids from Helda and Perona × Ouro Negro (HO and PO, respectively) after *M. incognita* and *M. javanica* infection. (**A**) Boxplots showing the mid-parent heterosis for all analyzed traits. (**B**) Boxplots showing the high-parent heterosis for all analyzed traits. Galling index (GI), number of egg masses per plant (EM).

The mid-parent heterosis (MPH) and high-parent heterosis (HPH) of GI and EM traits were assessed in F_1_ hybrids infected with *M. incognita* and *M. javanica* (Figure 3). Heterosis (MPH and HPH) values were mostly significantly different from the F_1_ mean value (Table S1). The results showed that the MPH values for GI ranged from 3.6 to 45.5%, whereas the HPH values varied from −4.4 to −34.6%. For EM, MPH ranged from 15.2 to 116.5%, and the mean values of HPH ranged from −30.3 to 25.9%.

Generally, the mean HPH values were lower than the MPH values for both traits, and positive MPH and negative HPH were observed, suggesting that the susceptibility in the hybrids tended to fall inside the parental range (Figure 3, Table S1). In addition, the F_1_ hybrid derived from the Helda parent had higher MPH and HPH values than the hybrid derived from Perona, while higher MPH and HPH values were observed in the comparison between isolates after infection with *M. incognita* than after infection with *M. javanica*. As compared to reproductive-related traits, GI showed lower MPH and HPH values in comparison with the EM trait.

**Figure 4:**
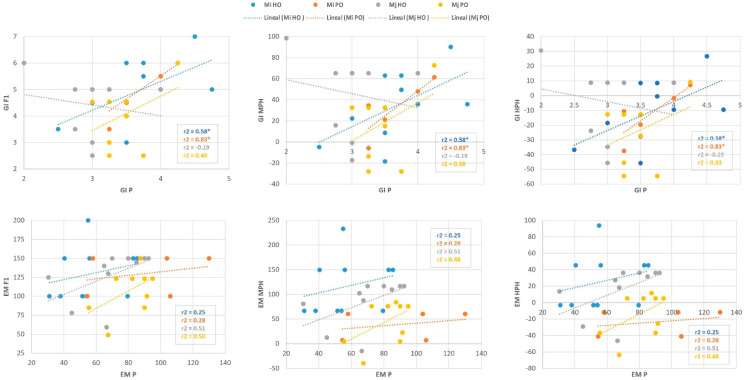
Correlation between parental performance, F_1_ performance, and heterosis. Helda and Perona × Ouro Negro crosses (HO and PO, respectively) for the *M. incognita* (Mi) and *M. javanica* (Mj) infection. Mid-parent heterosis (MPH), high-parent heterosis (HPH). Galling index (GI), number of egg masses per plant (EM). * indicates significant differences at *p* < 0.05.

Correlation analysis was carried out to investigate the effect of the performance of the parents on the performance of the hybrids (Figure 4). The results indicate that the correlation between the performance of the parental lines and the F1 generation, along with MHP/HPH, was only significant for the GI trait. This was observed for both hybrids (0.58 for HO and 0.83 for PO), in connection with the *M. incognita* isolate. This result suggests that genetic control of GI under *M. incognita* infection could be performed by additive genes, and the performance of parents can be used to predict hybrid performance with this isolate. However, the observed non-significant correlations across *M. javanica* infection and for the EM trait suggest that the transmission of this trait from the parental generation to the F_1_ generation may not follow a straightforward pattern of direct inheritance.

### 2.3. Inheritance Pattern of Resistance to Meloidogyne Derived from Ouro Negro

The mode of action (dominance or additivity) of the genes that govern the expression of GI and EM traits was analyzed using the dominance-to-additivity ratio (d/a); the data are summarized in Table S1. Both F_1_ progenies skewed segregations towards the susceptible parent. A d/a ratio of mostly partial dominance and overdominance was found in the HO and PO hybrids infected with *M. incognita* for both GI (0.69 and 0.63) and EM (1.79 and 0.58) traits, respectively. Under infection with *M. javanica* partial dominance and overdominance were detected for EM trait for HO and PO hybrids (1.76 and 049, respectively).

**Figure 5:**
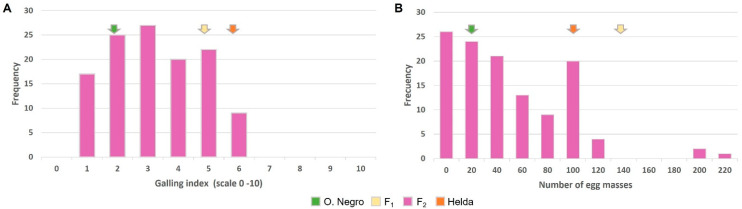
Frequency distribution of the resistance to *M. incognita* of F_2_ families from the Helda × Ouro Negro cross. (**A**) Galling index. (**B**) Egg mass number. The mean values of both parents and F_1_ are shown in different colors.

In order to assess whether the inheritance of the resistance to *M. incognita* in Ouro Negro is either monogenic or quantitative, resistance phenotyping was carried out on F_2_ HO hybrid populations (Table 1). The values of the F_2_ families were not normally distributed, as confirmed by a Shapiro–Wilk test for GI (W = 0.95, *p*-value = 0.0001) and EM (W = 0.90, *p*-value = 0.0001) traits. The distribution was skewed toward susceptibility. Box–Cox transformation resulted in normality for both traits. In the F_2_ resistance assessment assay (Figure 5A), the average GI (±SD) values of *M. incognita* infections for F_1_, Helda and Ouro Negro were 5.0 ± 1.58 (ranging from 3 to 7; n = 5), 5.5 ± 1.14 (ranging from 3 to 8; n = 19), and 1.9 ± 0.61 (ranging from 1 to 3; n = 55), respectively. The maximum GI observed in the Ouro Negro parental donor (GI = 3) was used as a cut-off value to determine the threshold below which F_2_ progenies could be considered resistant. Likewise, F_2_ plants were considered susceptible with a GI ≥ 3. The segregation ratio of resistant and susceptible plants (R:S) was then calculated based on the full dataset (with GI ranging from 1 to 6.5 across 120 F_2_ plants; Figure 5A). The observed segregation ratio for F_2_ deviated significantly from the ratios of 1:3 (χ2 = 6.40, *p* = 0.01141), 1:15 (χ2 = 169.28, *p* < 0.00001), and 1:63 (χ2 = 872.30, *p* < 0.00001) (Table S2), which is expected as the resistance depends on one, two, or three independent recessive resistance genes. Chi-square test analysis instead indicated that the resistance in F_2_ followed a segregation ratio of 7:9 (χ2 = 3.73, *p* = 0.053) (Table S2), which indicates that two genes were involved in the quantitative control of nematode resistance following a double dominant–recessive epistasis interaction. Accordingly, the minimum number of involved genes for the GI trait was estimated to be about 2.5 to 3.3; a value of 0.62 indicates a relatively high heritability (Table S3).

In the F_2_ resistance assessment assay (Figure 5B), the average EM (±SD) of *M. incognita* on F_1_, Helda, and Ouro Negro plants was 130 ± 44.7 (ranging from 100 to 200; n = 5), 103 ± 38.1 (ranging from 50 to 150; n = 19), and 21 ± 14.9 (ranging from 2 to 50; n = 55), respectively. The maximum EM observed in the Ouro Negro parental donor (EM = 50) was used as a cut-off value to determine the threshold below which F_2_ progenies were considered to be resistant. Likewise, F_2_ plants were considered susceptible with an EM > 50. The segregation ratio was calculated on the full dataset (with EM ranging from 2 to 223 across 120 F_2_ plants; Figure 5B). The F_2_ plants exhibited a segregation ratio of 7R:9S (χ2 = 1.90, *p* = 0.16755; Table S2), again confirming the existence of two genes interacting in a dominant–recessive epistatic model responsible for the control of nematode reproduction. Thus, the hypothesis based on the previous analysis of the segregation was confirmed. The minimum number of genes involved in the EM trait was estimated to be about 1.9 to 5, and the value of 0.54 indicates relatively high heritability (Table S3).

The means and segregation ranges of the F_3_ progenies (families 20, 101, and 102) derived from single resistant F_2_ plants showed no gall development (ranged from 1 to 3) and highly reduced egg mass production (ranged from 2 to 51) for most of the descendant plants (Table S4), which provides evidence for the successful transmission of resistance genes from Ouro Negro to valuable common bean germplasm. (Table S4). Correlation analysis between GI and EM responses was performed using data from the F_2_ population, and these traits were highly correlated (r = 0.75, *p* = 0.0001). In summary, heritability values assessed for RKN reproduction, i.e., GI and EM, were quite similar after infection with two different nematode species, which is consistent with the genetic nature of the resistance provided by the Ouro Negro cultivar.

## 3. Discussion

Root-knot nematodes (RKN) resistance is controlled by dominant [13,32,39] or recessive [10,31,33,40,41] genes, or by additive expression. This resistance can be conferred by single major genes, combinations of two or more genes, or quantitative trait loci (QTLs) [42]. Despite progress, achieving sustainable RKN resistance remains a significant challenge. Combining multiple favorable alleles from diverse sources of RKN tolerance into a single hybrid is a critical hurdle for the long-term production of common bean crops globally. This study further demonstrates that that the resistance of Ouro Negro can be successfully transferred into elite common bean cultivars.

The *M. incognita* and *M. javanica* resistance response was examined in populations derived from the crossing between two snap bean accessions (the susceptible Helda and Perona female parents) and the Ouro Negro cultivar (the resistant male parent). In this study, responses to nematode infections were analyzed by assessing the number of egg masses and the root-galling index, two parameters that define the susceptible/resistant phenotype very well. In hybrid breeding programs, the most important and difficult task is the selection of parental lines based on the predicted performance of the hybrid itself. Systematic surveys on the heterosis of agronomic and stress resistance characteristics have been performed in several crop species, which evidenced that the magnitude of heterosis is highly dependent on the parental lines and the trait(s) measured [43,44,45,46,47]. Significantly negative HPH and positive MPH values for the egg mass number were observed in the F_1_ hybrids for infection with both isolates (Table S1). The negative HPH value indicates that the F_1_ hybrid has a superior resistance to nematodes compared to its susceptible parent, and the inbred lines featured genes related to *Meloidogyne* resistance. Moreover, the high positive correlation between the mean parent and hybrid performance observed for the galling index in response to *M. incognita* indicates that it is a good indicator for selecting nematode-resistant sources as parents (Figure 4). This correlation was non-significant for the number of egg masses, which is anticipated, as it is a complex trait influenced by dominant masking effects. In addition, high correlations between root-galling and nematode reproduction phenotypes and between *M. incognita* and *M. javanica* responses were observed in this study, which suggests that both traits might be controlled by dependent genetic mechanisms and that the same genes are effective for both isolates. Similarly, a significant correlation between root-galling and reproduction phenotypes was reported in cowpea recombinant inbred populations [42]. However, a differential response to root-galling and reproduction has been reported in leguminous crops such as the common bean [48,49], soybean [50], and lima bean [31]. Consequently, the suitability of a common bean host (based on galled roots and nematode reproduction) for one nematode species may change depending on the genotype, or character, of the nematode interaction. This is a highly specialized nematode–plant interaction where the abundance of galled roots suggests that nematodes have invaded the tissue, although this does not necessarily imply that they will be capable of laying eggs or sufficient reproduction.

The fact that all of the F_1_ plants were highly susceptible to *M. incognita* and *M. javanica* and not significantly different from the susceptible parents, as well as the fact that the mid-parent performance was mostly substantially lower than the mean of the F_1_ generation, suggests partial to overdominant inheritance in favor of recessive resistance. Moreover, we were able to study the segregation of *M. incognita* resistance in the F_2_ population resulting from self-fertilization of F_1_ individuals. The observed segregation ratios were 7R:9S for both analyzed GI and EM traits. The 7R:9S ratio observed in F_2_ from the Helda × Ouro Negro cross confirmed the existence of two unlinked, complementary genes and suggests that the recessive alleles of each gene resulting from the Ouro Negro cultivar are required to confer full resistance to *M. incognita*. The present results are complementary to the findings in other bean—*Meloidogyne incognita* interactions, with researchers reporting that phenotypic resistance for the number of egg masses in the PI165426 accession is under the control of one to two genes (one dominant and one recessive gene (*Me2* and *me3*) due to a transition from a dominant to a recessive system caused by temperature [32]. However, this two-gene hypothesis is not sufficient to explain the segregation patterns observed in the galling index [10], and hence, at least three or more genes would be needed to determine nematode resistance. Similar to our results, two resistance genes, *Rk1* and *Rk2*, were identified in cowpea, conferring partial dominant resistance against RKN [42]. In soybean, the resistance of the cultivar Peking is suggested to be bigenic, involving *rhg1-a* and *Rhg4* [51]. In addition, the gall-free and egg mass production observed in some individuals in the F_2_ progenies analyzed here, as well as the possibility that these plants have a low amount of galling and number of egg masses due to a low level of inoculum or phenotyping errors, was disregarded given the drastic reduction in galling and egg production showed by the F_3_ offspring (Table S4). The closeness of the F_3_ mean to the value of the resistant F_2_ individuals also confirms the efficiency of the inheritance of nematode resistance.

The estimates of heritability of resistance observed for root-galling and egg masses in the F_2_ generation in response to *M. incognita* were in the range of 0.62–0.54, indicating again that the resistance in Ouro Negro can be transferred successfully into elite common bean cultivars. This is important because the greater susceptibility to nematode infection observed in our populations can arise as an effect of intensive crop breeding, which evidences the lack of disease resistance and adaptive alleles [52]. A previous study [53] demonstrated that domesticated common bean accessions had a lower natural resistance than the wild accessions against bacterial pathogens. One proposed explanation is that RKN resistance has decreased in the cultivars produced during the modern era of plant breeding, making plants highly susceptible to pathogens, pests, and environmental changes [54]. Therefore, less intensively improved varieties are often used to identify novel sources of resistance against diverse pathogens, which highlights the importance of the resistant genetic resources identified here. The identification of two stable resistance genes from the Ouro Negro cultivar in this study provides a foundation for further functional analysis and paves the way for utilizing this resistance trait. The development of DNA markers that co-segregate with these genes will be a powerful tool and could be routinely used in common bean breeding programs.

## 4. Materials and Methods

### 4.1. Plant Material

The cultivar Ouro Negro (ON) used in the present study was previously described to be resistant to *M. incognita* and *M. javanica* [14,17,35,55,56]. The snap bean varieties Helda (H) and Perona (P) were used as susceptible varieties (Figure 1A). The Phy5, Phy90, PhyIM (susceptible), and Aporé (resistant) cultivars were used as controls. Crosses were made between the resistant male donor variety (Ouro Negro) with a susceptible female donor variety, either Helda or Perona, in order to obtain F_1_ seed (designated as F_1_ HO and PO, respectively). F_1_ hybrid plants are the result of crossing the two pure-parent lines that have been developed by inbreeding to have consistent characteristics from one generation to the next. F_2_ progeny was obtained from the self-pollination of the F_1_ HO hybrid (designated as F_2_ HO). Each F_2_ plant was handled separately to generate F_3_ progenies, and a total of three F_3_ families (57 plants) were then screened in pouches for their response to *M. incognita*. Table 2 provides an overview of the experiments carried out, where Experiments (Exp.) 1 and 2 correspond to *M. incognita* and *M. javanica* infection, respectively, of Helda, Perona and Ouro negro parents, F_1_ HO and PO, and Aporé, Phy5, Phy90 and PhyIM controls; Experiment 3 was carried with *M. incognita* infection on F_2_ HO, F_1_ HO, and Helda and Ouro Negro parents; and Experiment 4 have been conducted with *M. incognita* on F_3_ HO.

### 4.2. Nematode Isolates and Resistance Phenotyping Evaluation

Pure cultures of *M. incognita* race 2 and *M. javanica* collected in the Mediterranean area (southwestern Spain, Almería) were used in this study. Both isolates were obtained from a single egg mass and were identified via a differential host test [57]. These nematode isolates were multiplied separately on a tomato (*Lycopersicon esculentum*) cultivar, Marmande Claudia. The Baermann funnel technique was used to extract the second-stage juveniles (J2) of each *Meloidogyne* spp. isolate from roots and soil. The juveniles were stored at 4 °C for a maximum of 3 days before inoculation [58].

Seeds were germinated in pouches in the absence of soil, and the seedling growth pouch was 15.5 × 12.5 cm and made of a paper wick. It was folded at the top to form a 2 cm deep trough in which the seed or seedling was placed. The paper wick was contained inside a transparent plastic pouch. The seedling growth pouches were placed in hanging folders in a growth chamber at 23–24 °C and a relative humidity of 45–60% and 85–100% during the light/dark period, respectively, with a light cycle of 14 h [59]. The growing plants were arranged in a completely randomized design and watered lightly three times a week and with a commercial nutrient hydroponic solution once a week. At 10–15 days after germination (after the development of two true leaves and the root system), seedlings were individually inoculated with 500 J2s delivered in 5 mL aqueous suspensions, distributed directly over the roots with a pipette tip. Pouches were maintained in a horizontal position for 24 h after inoculation, covered with dark paper to protect from light, and then returned to hanging folders in the growth chamber under the same experimental conditions (Figure 1B). Forty-five days after inoculation (before completion of the second cycle of nematode reproduction), each pouch was infused with about 10–20 mL of 75 mg/L erioglaucine to color the egg masses green [60]. The pouches were flooded with the dye in a horizontal position overnight (Figure 1C). After staining, the pouches were drained and the root systems were evaluated by counting the egg masses under an illuminated Nikon SMZ25 stereomicroscope (Nikon, Japan) at 20× magnification. The growth pouches allow direct observation of nematode infection symptoms, root-galling, and egg mass production under their transparent surfaces. Nematode reproduction was evaluated using the root-galling index (GI) using a 0 to 10 scale based on the percentage of the root system with galls and their distance to the main root, where 0 = no gall; 1–2 = trace infection with 1–4 small galls; 3–4 = 5–10 galls in the roots; 5 = 50% roots galled; and 10 = 100% roots galled or a dead plant [61]. Eggs were extracted from root systems separately, and the number of egg masses per plant (EM) was counted, corresponding to the number of J2 able to complete their life cycle. Plants with a GI < 3 were classified as RKN resistant, and those with a GI ≥ 3 were classified as susceptible [62]. The EM was used as a parameter of plant resistance, where plants with ≤50 egg masses were proposed to be resistant to nematode isolate infection.

### 4.3. Genetic Analyses of RKN Resistance: Heterosis, Inheritance and Allelism Test

The quantitative data from the genotype × nematode isolate experiments were subjected to an analysis of variance using the XLSTAT statistical package [63]. Prior to the analyses, GI and EM parameters were tested for normality, and Box–Cox transformations were used where appropriate to transform the data. The significance of differences between common bean genotypes and/or *Meloidogyne* isolates was tested using Tukey’s honestly significant difference (HSD) test at the 95% confidence level.

The heterosis of F_1_ hybrids (hybrid vigor) was computed for *Meloidogyne* spp. under the experimental conditions. MPH and HPH were calculated using the following formulas: MPH = [F_1_ − (P_1_ + P_2_)/2]/[(P_1_ + P_2_)/2] × 100% and HPH = (F_1_ − HP)/HP × 100%, where F_1_ is the value of F_1_ hybrids, P_1_ and P_2_ are the phenotypic value of parents, and HP is the phenotypic value of the high parent. The heterosis of F_2_ was calculated as HF_2_ = (2F_2_ − P_1_ − P_2_)/(P_1_ + P_2_). Pearson’s correlation coefficient (r) was used to analyze the correlation between the performance of the parents and the F_1_ generation and heterosis (MPH and HPH), tested at *p* = 0.05 and 0.01.

To determine the mode of inheritance of quantitative resistance (additive or dominant), the GI and EM values of F_1_ hybrids were compared to the average value of their parental lines (MP, mid-parent values). An additive effect was defined as a = (Trait_parent1_ − Trait_parent2_)/2, and a dominant effect was defined as d = Trait_F1_ − (Trait_parent1_ + Trait_parent2_)/2. Genetic effects were classified by the value of the dominance/additivity ratio (or d/a ratio), with additive effects when d/a < 0.2, partially dominant effects when 0.2 ≤ d/a < 0.8, dominant effects when 0.8 ≤ d/a ≤ 1.2, and overdominant effects when d/a > 1.2 [64].

Chi-square tests were used to determine the goodness-of-fit from observed to expected segregation ratios in the F_2_ generation for GI and EM. For each phenotypic trait, variance components and broad-sense heritabilities (h^2^) with their standard errors were estimated by the restricted maximum likelihood (REML) option of the PROC MIXED and IML procedures (SAS Institute Inc. v. 9.04, Cary, NC, USA) [65]. The minimum number of genes controlling resistance was quantitatively estimated using the following methods: (i) Wright’s method [66]: (µ_P1_ − µ_P2_)2 × {1.5 − [2 × (µ_F1_ − µ_P1_/µ_P2_ − µ_P1_) × (1 − (µ_F1_ − µ_P1_/µ_P2_ − µ_P1_))]}/8 × [δ^2^_F2_ − {δ^2^_P1_ + δ^2^_P2_ + (2 δ^2^_F1_)}/4]; (ii) Lande’s method I [67]: (µ_P1_ − µ_P2_)2/8 × [δ^2^_F2_ − {δ^2^_P1_ + δ^2^_P2_ + (2 δ^2^_F1_)}/4], where µ_P1_, µ_P2_, and µ_F1_ refer to the means of parent 1 and 2 and F_1_, respectively, and δ^2^_F2_, δ^2^_P1_, and δ^2^_P2_ refer to the variances of F_2_, P_1_, and P_2_, respectively.

## 5. Conclusions

In summary, we propose a duplicate recessive epistatic pattern as the genetic inheritance mechanism for the RKN resistance provided by the Ouro Negro common bean cultivar against *M. incognita* and *M. javanica*. These results highlight the importance of understanding the sources of genetic resistance to RKN and provide a valuable reference for future mapping approaches. This will facilitate the development of closely linked molecular markers for these two genes, as well as the pyramid combinations of natural resistance genes, enabling the creation of broad-based and durable root-knot nematode resistance in common bean.

## Figures and Tables

**Table 1:** Average and standard error (S.E.) of galling index and number of egg masses per plant for each common bean genotype, F_1_ hybrid, and *Meloidogyne* isolate.

Genotype	GI ^1^				EM			
	*M. incognita*		*M. javanica*		*M. incognita*		*M. javanica*	
	Mean	S.E	Mean	S.E	Mean	S.E	Mean	S.E
Ouro Negro	1.88	0.08	1.82	0.25	22.15	2.02	31.86	3.84
Aporé	1.44	0.19	0.83	0.22	28.00	4.44	7.89	2.03
PhyIM	3.30	0.26	4.64	0.36	128.57	10.10	132.86	11.28
Phy90	4.91	0.24	Ne	Ne	147.18	23.25	Ne	Ne
Phy5	6.05	0.12	Ne	Ne	103.10	10.05	Ne	Ne
F_1_ HO	4.95	0.39	4.40	0.35	130.00	11.06	127.70	10.35
F_1_ PO	4.90	0.43	3.60	0.66	130.00	12.25	93.80	16.37
Helda	5.10	0.24	4.60	0.34	99.48	8.74	110.00	12.47
Perona	5.60	0.46	5.50	0.13	170.00	30.00	134.50	5.98

^1^ Galling index (GI), number of egg masses per plant (EM). Ne = not evaluated. HO = Helda × Ouro Negro cross, PO = Perona × Ouro Negro cross.

**Table 2:** Summary of pot experiments for the multiplication of different nematode species on common bean genotypes under seedling growth-pouch conditions.

Genotype	No. Plants ^a,b^			
	Exp. 1	Exp. 2	Exp. 3	Exp. 4
	*M. incognita*	*M. javanica*	*M. incognita*	*M. incognita*
Helda parent	5	10	20	
Perona parent	5	10		
F_1_ Helda × Ouro Negro line	7	10	6	
F_1_ Perona × Ouro Negro line	5	4		
Ouro Negro parent	18	8	27	
Aporé resistant control	8	9		
Phy5 susceptible control	11			
Phy90 susceptible control	12			
PhyIM susceptible control	8	15		
F_2_ Helda × Ouro Negro lines			121	
F_3_ Helda × Ouro Negro lines				57

^a^ Exp. = Experiment. ^b^ M = *Meloidogyne*.

## Data Availability

The data presented in this study are available on request from the corresponding author.

## Supplementary Materials

The following supporting information can be downloaded at https://www.mdpi.com/article/10.3390/plants14071073/s1: Table S1: The mean values and standard errors of F_1_, mid-parent (MP), mid-parent heterosis (MPH), high-parent heterosis (HPH), and estimations of the dominance-to-additivity ratio for *M. incognita* and *M. javanica* incidence under experimental conditions for Helda × Ouro Negro (HO) and Perona × Ouro Negro (PO) crosses; Table S2: Chi-square test analysis of the segregation ratios for resistance to *M. incognita* in the F_2_ population from a susceptible Helda × resistant Ouro Negro cross. Table S3: Minimum number of effective factors and heritability from an F_2_ population developed from a Helda × Ouro Negro cross; Table S4: A comparison of *M. incognita* resistance of F_3_ families of the Helda × Ouro Negro cross.
